# Housekeeping in Tephritid insects: the best gene choice for expression analyses in the medfly and the olive fly

**DOI:** 10.1038/srep45634

**Published:** 2017-04-03

**Authors:** Efthimia Sagri, Panagiota Koskinioti, Maria-Eleni Gregoriou, Konstantina T. Tsoumani, Yiannis C. Bassiakos, Kostas D. Mathiopoulos

**Affiliations:** 1 Department of Biochemistry and Biotechnology, University of Thessaly, Larissa, Greece; 2 Department of Economic Sciences, National and Kapodistrian University of Athens, Athens, 10559, Greece

## Abstract

Real-time quantitative-PCR has been a priceless tool for gene expression analyses. The reaction, however, needs proper normalization with the use of housekeeping genes (HKGs), whose expression remains stable throughout the experimental conditions. Often, the combination of several genes is required for accurate normalization. Most importantly, there are no universal HKGs which can be used since their expression varies among different organisms, tissues or experimental conditions. In the present study, nine common HKGs (*RPL19, tbp, ubx, GAPDH, α-TUB, β-TUB, 14-3-3zeta, RPE* and *actin3*) are evaluated in thirteen different body parts, developmental stages and reproductive and olfactory tissues of two insects of agricultural importance, the medfly and the olive fly. Three software programs based on different algorithms were used (*geNorm, NormFinder* and *BestKeeper*) and gave different ranking of HKG stabilities. This confirms once again that the stability of common HKGs should not be taken for granted and demonstrates the caution that is needed in the choice of the appropriate HKGs. Finally, by estimating the average of a standard score of the stability values resulted by the three programs we were able to provide a useful consensus key for the choice of the best HKG combination in various tissues of the two insects.

The Mediterranean fruit fly, *Ceratitis capitata* (Wiedemann), and the olive fruit fly, *Bactrocera oleae* (Rossi), belong to the Tephritidae family of insects. As typical fruit flies, females lay their eggs in fruits or vegetables and the emerging larvae feed in the fruit sap, thus destroying the fruit. The medfly is one of the most devastating insects, easily adapting to new environments and hosts, infecting more than 260 species of fruits and vegetables worldwide^1^,^2^, and causing great economic losses in fruit production and quarantine costs. The olive fruit fly, on the other hand, is a monophagous species, the most important enemy of olive cultivations^3^,^4^. Whole genome sequencing of both species has been completed^5^,^6^, offering a holistic view of the entire genomes, allowing the study of any desired gene and thus leading to a profound understanding of the biology of these species. Such understanding is a prerequisite for novel, alternative to insecticides, control approaches.

The study of any gene inevitably goes through detailed and thorough scrutiny of its expression profile in various tissues and under different conditions. An invaluable tool for such expression analysis is RT-qPCR. The same way PCR revolutionized modern day molecular biology, RT-qPCR gave tremendous impetus to studies of gene expression, quantitative genotyping, genetic variation, disease diagnosis, forensics and many more. Due to the simplicity of the reaction, data can be easily collected and published in high impact journals without, necessarily, following good practices of RT-qPCR^7^. One of the most important parameters that should be addressed in order to standardize the reaction and perform a valid RT-qPCR analysis is the selection of suitable reference housekeeping genes. Since the reaction has several limitations as a result of the quality and quantity of starting RNA and the efficiency of its reverse transcription, housekeeping genes are used for the systematic normalization of gene expression data in order to improve the fidelity and accuracy of RT-qPCR^8^,^9^,^10^. Time and again, it has been demonstrated that the use of an unsuitable reference gene can lead to false results of the qPCR data and, consequently, to erroneous interpretations^11^,^12^,^13^,^14^. Most frequently, indeed, more than one housekeeping genes are required for proper normalization of the data^15^,^16^.

In insects, many articles have been published on the identification and selection of the best reference gene in specific tissues and under different conditions. In the Tephritidae family there are two studies on the oriental fruit fly, *Bactrocera dorsalis*^17^,^18^ and one in the West Indian fruit fly *Anastrepha obliqua*^19^. Among other dipteran species, there are three studies on *Drosophila melanogaster*^15^,^20^,^21^ and a single one on each of *D. suzukii*^22^, *Musca domestica*^23^, *Lucilia cuprina*^24^ and the Calliphoridae family^25^. Interestingly, there are no studies published on any mosquito species. In many mosquito publications, normalization of RT-qPCR is at best performed using a housekeeping gene (HKG) that demonstrates stable expression in microarray or RNAseq results^26^,^27^. This strategy may seem biologically reasonable, but there is a potential technical artifact considering that microarrays, RNAseq and RT-qPCR constitute quite different methods, with different limitations, requiring different standardization each. Most frequently, however, there is no specific justification regarding the selection of the utilized HKGs^28^,^29^,^30^,^31^,^32^,^33^,^34^ except, at most, that it may have been used previously in the same^35^,^36^ or related species^37^,^38^. Furthermore, with regard to published HKG studies on Diptera, the number of HKGs tested varies from as low as six^18^,^21^,^25^ to over 20^15^. Unfortunately, neither the same genes nor the same tissues and conditions are studied, a fact that makes any effort to compare results practically impossible. Very importantly, these studies hardly ever indicate the use of the same housekeeping gene or gene combination in different tissues of the same insect or in the same tissue of different insects. Since, as mentioned above, the use of improper housekeeping genes for the normalization of the RT-qPCR can lead to erroneous results, this variability necessitates each time, for every organism and every tissue, the search for the proper housekeeping genes. Additionally, given the fact that the available software (such as *geNorm*^9^, *NormFinder*^39^, *BestKeeper*^40^ *and* the web-based *RefFinder* platform^41^) are based on different statistical algorithms, they do not result in the same HKG suggestions for a particular tissue^42^,^43^.

Here we present the most extensive study on HKGs, at least in the dipteran order of insects. The study validates nine candidate reference genes in thirteen different tissues of the model tephritid fly, the Mediterranean fruit fly, *C. capitata,* and the olive fruit fly, *B. oleae*. The genes are: *RPL19 (ribosome protein L19*), *tbp (TATA-binding protein*), *ubx (ultrabithorax*), *GAPDH (glyceraldehyde 3-phosphate dehydrogenease*), *α-TUB (α-tubulin*), *β-TUB (β-tubulin*), *14-3-3zeta, RPE (RNA polymerase II*) and *actin3.* The tissues selected for the analysis were mostly tissues from either the reproductive [testes, ovaries, male and female accessory glands (MAGs and FAGs, respectively), ovipositors] or the olfactory (maxillary palps and antennae) systems of the flies. In addition, we analyzed three developmental stages (egg, larva, pupa) and the three sections of the insect body (head, thorax, abdomen), as they are often convenient controls for comparison with other tissues.

## Results

In the present study, the best choice for reference genes for RT-qPCR in thirteen tissues of two insects of the Tephritidae family, the Mediterranean fruit fly, *Ceratitis capitata* and the olive fruit fly, *Bactrocera oleae*, was examined. Three available software programs were used for the analysis and, since each program is based on a different algorithm, an effort was put to generate a consensus of the three programs.

### Gene choice and amplification performance

Nine different housekeeping genes, commonly used in other dipteran species, were chosen for the analysis. The genes considered were: *RPL19 (ribosome protein L19*), *tbp (TATA-binding protein*), *ubx (ultrabithorax*), *GAPDH (glyceraldehyde 3-phosphate dehydrogenease*), *α-TUB (α-tubulin*), *β-TUB (β-tubulin*), *14-3-3zeta, RPE (RNA polymerase II*) and *actin3*. Gene names and IDs for the two species are presented in Supplementary Table S6.

In all instances, primers were designed by Primer-BLAST^44^ in order to get amplicons ranging from 82 to 150 bp, as shown in Supplementary Table S7. Reaction conditions described in the Methods section resulted in one gene-specific peak and the absence of primer dimers peaks (data not shown). The PCR efficiency (E) and the correlation coefficient (R^2^) characterizing each standard curve are also given in Supplementary Table S7. Efficiencies for all tested genes varied between 90.1% and 106.4%.

All reactions were done in triplicate (three technical replicates). The expression of the reference genes was measured in 8 or 10 biological replicates, as indicated in Table 1. Three negative controls were also used.

### Expression stability by *geNorm*

**Figure 1:**
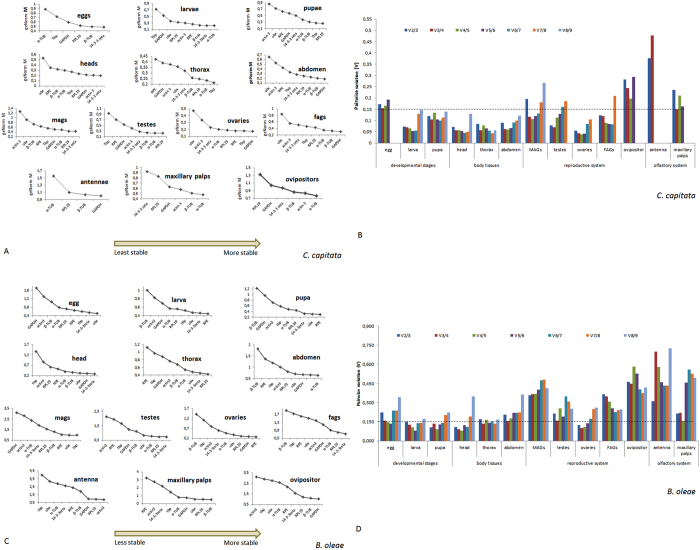
(**A**) Stability values of the reference genes in the 13 *C. capitata* tissues under study as generated by the *geNorm* algorithm. The average expression stability values from least stable (left) to most stable (right) for the egg, larva, pupa, head, thorax, abdomen, MAGs, testes, ovaries, FAGs, antennae, maxillary palps and ovipositor of the Mediterranean fruit fly. (**B**) Pairwise variation (V) of the housekeeping genes computed by *geNorm* in *C. capitata*. The pairwise variation (V_n_/V_n+1_) analysis determines the optimal number of reference genes for all of the tissues under study. (**C**) Stability values of the reference genes in the 13 *B. oleae* tissues as generated by the *geNorm* algorithm. The average expression stability values from least stable (left) to most stable (right) for the egg, larva, pupa, head, thorax, abdomen, MAGs, testes, ovaries, FAGs, antennae, maxillary palps and ovipositor of the olive fruit fly. (**D**) Pairwise variation (V) of the housekeeping genes computed by *geNorm* in *B. oleae*. The pairwise variation (V_n_/V_n+1_) analysis determines the optimal number of reference genes for all of the tissues under study.

*geNorm* is a Visual Basic Application (VBA) for Microsoft Excel that automatically calculates two parameters: the gene-stability measure M and the pairwise variation V. The lower the gene-stability M value indicates the more stably expressed gene. Values of M higher than 1.5 are not considered stable across measurements. The pairwise variation V, on the other hand, indicates the least number of the most stably expressed genes that should be combined for optimal normalization. Additionally, V should be below the cut-off value of 0.15, otherwise, the lowest V should be considered. Using this algorithm, we ranked the nine housekeeping genes in the thirteen tissues tested according to their expression stability (Fig. 1). For *B. oleae* egg, for example, under the cut-off value of 0.150 is V4/5 (0.136, Fig. 1-D) and, therefore, the four most stable genes for the eggs (*ubx* with M = 0.508*, 14-3-3zeta* with M = 0.556*, tbp* with M = 0.601 and *RPE* with M = 0.658) should be combined in order to obtain optimal normalization. For the other tissues, the lowest pairwise variation value and the suggested combination of HKGs are presented in Supplementary Table S1. In most cases, *geNorm* suggests the combination of 2–3 HKGs for optimal normalization. In one case (FAGs of *B. oleae*) it suggests the combination of six; and in one other (ovipositor of *B. oleae*) it suggests the combination of seven. For *C. capitata, α*- and *β-tubulin* are most frequently among the suggested HKGs, while for *B. oleae 14-3-3zeta* is the winner. *RPE* and *ubx* are never among the suggested HKGs in *C. capitata*.

### Expression stability by *NormFinder*

*NormFinder* algorithm identifies the optimal normalization gene among a set of candidate genes, providing a stability value for each gene. This value is the estimated expression variation if a given gene is used for normalization. Therefore, the candidate genes can be ranked according to their expression stability in the different tissues or experimental conditions^45^. The calculated stability values for each HKG and the according ranking in the thirteen tissues are shown in Supplementary Tables S2A and S2B for *C. capitata* and *B. oleae*, respectively. For *C. capitata, GADPH* ranks first in four tissues, *14-3-3zeta* in three, while *ubx* and *actin3* never rank first. For *B. oleae, RPE* and *14*-*3-3zeta* rank first in three tissues each, while *ubx* and *GADPH* never rank first.

### Expression stability by *BestKeeper*

*BestKeeper* software estimates standard deviation (SD) of the Ct values of all candidate genes. Since the expression levels of suitable HKGs should be highly correlated, the lower the SD the more stable the gene^40^. The disadvantage of *BestKeeper* is that it does not provide a combination of reference genes required for an experiment. The calculated SD values and CV (coefficient of variation) for each HKG in the thirteen tissues are shown in Supplementary Tables S3A and S3B for *C. capitata* and *B. oleae*, respectively. According to *BestKeeper, α-TUB, GADPH* and *RPL19* have the least SD values in three different tissues of *C. capitata* each, while *tbp* and *ubx* in none. For *B. oleae, α-TUB* and *RPE* have the least SD in four different tissues each, while *β-TUB, ubx, GADPH* and *actin3* in none.

### Seeking consensus

Since the different software programs use different algorithms to estimate gene expression stability, they rarely reach the same ranking. *RefFinder* software theoretically integrates the results of the previous analyses (by *geNorm, Normfinder* and *BestKeeper*). It then assigns an appropriate weight to an individual HKG and calculates the geometric mean of their weights for an overall final ranking^41^. We ran this user-friendly web-based tool as well. However, since the values that *RefFinder* calculated for, e.g., *geNorm* differed from those estimated by *geNorm* itself, we considered *RefFinder* unreliable and we did not use it any further. *RefFinder* results are presented in Supplementary Tables S4A and S4B.

In order to propose a combination of the most stable HKGs that a researcher can use for normalization of gene expression in *C. capitata* and *B. oleae*, we took a different route. We first estimated the average of a standard score (z-score) of the stability values resulted by all three software packages for every single gene and then ranked them according to this new average score. Complete results of this ranking are presented in Supplementary Tables 5A and 5B. The first three genes of this consensus ranking are presented in Table 2. In the medfly, *RPL19* is the HKG that is most often found in the best three ranking genes, followed by *β-TUB*, while *ubx* is never among the top three. Similarly, in the olive fly *14-3-3zeta* is the HKG that is most often found in the best three ranking genes, followed by *GADPH*, while *α-TUB* is not found at all. To our experience, the combination of at least two HKGs and at most the number of genes suggested by *geNorm*, provides an excellent internal control in all RT-qPCRs.

## Discussion

Several times in the recent years it has been documented that the choice of the right reference gene/s for the standardization of RT-qPCRs is of paramount importance and the possible use of the inappropriate HKGs can lead to incorrect results^11^,^12^,^13^,^14^. Common housekeeping genes, that are supposed to be constitutively expressed in order to maintain basic cellular functions, may not have constant and stable expression throughout an experiment. This may be due to the special characteristics of the organism or tissue analyzed or the particular conditions of the experimental design. Good practice of an RT-qPCR experiment requires the establishment of the appropriate HKGs for its standardization^46^, even though good practice is not always observed.

We set out to address the above question for the medfly, *C. capitata*, and the olive fly, *B. oleae*, both very important agricultural pests. Particularly, the medfly is a cosmopolitan pest and due to its great importance in the cultivation and export of more than 260 fruits and vegetables^1^,^2^, it has turned out to be a model organism in the Tephritidae family of insects and beyond, for studies ranging from classical genetics to genomics^5^,^47^,^48^,^49^,^50^,^51^,^52^, as well as area-wide control practices^53^,^54^. The olive fly, on the other hand, is a strictly monophagous cousin of the medfly, of particular interest in the olive producing areas of the world^4^. Recent development of molecular and genomics tools have made it focus of active research, with renewed interest in its control^55^,^56^. The tissues selected for the analysis were mostly tissues from either the reproductive (testes, ovaries, male and female accessory glands, ovipositors) or the olfactory (maxillary palps and antennae) systems of the flies. The reproductive system is involved in the successful mating and egg development while the olfactory system plays a crucial role in insect survival and reproductive success, mediating responses to food, mates and oviposition. Beyond their general interest, such systems can serve as targets for alternative control approaches, such as the Sterile Insect Technique and its alternatives^52^,^57^,^58^,^59^ and, therefore, are currently under scrutiny in the scientific community. In addition, we analyzed three developmental stages (egg, larva, pupa), as they are useful in order to obtain the expression profile throughout the life cycle of an insect. Finally, we included the three sections of the insect body (head, thorax, abdomen), as they are often convenient controls for comparison with other tissues.

In order to determine the best combination of HKGs, we performed our analyses with the three most popular software programs, *geNorm*^9^, *NormFinder*^39^ and *BestKeeper*^40^. As anticipated, results were largely inconsistent among them, as they are based on different algorithms. A fourth user-friendly web-based software, *RefFinder*^41^, that is supposed to integrate the results of the previous three programs, gave inconsistent results with the programs themselves and so it was deemed untrustworthy. Instead, we decided to take a different route: we first transformed the raw scores of the three programs into standard scores, then calculated the average of the standard scores and finally ranked them. The use of the average of scores is based on the underlying idea of producing a composite score using a linear combination of the individual score values. This practice is common in Statistics, e.g., Principal Components Analysis, Factor Analysis and other multivariate^60^,^61^,^62^ methods. In all these methods the individual variables do not have to be similar in derivation nor do they have to measure the same quantity. Instead, they measure different facets of the same concept, in many instances using different measurement tools. This is the case in our work. Averaging is the simplest form of linear combination (all scores have the same coefficient).The major issue in this case is not how the individual scores are derived, but if their values are in a similar range. When this is not true the score with the larger values would dominate the composite score. We resolved this problem by score standardization.

The aforementioned approach resulted in a useful consensus key (Table 2) for the choice of the best HKG combination in various tissues of the medfly and the olive fly. A few qualified comments based on Table 2 are worth making. First, the most common genes found in the top three choices for both the medfly and the olive fly (i.e., found five times or more in both organisms in Table 2) are *14-3-3zeta, RPL19* and *GAPDH*, while the least common (two times or less) are, *tbp* and *ubx*. Curiously, *α-* and *β-tubulins* are quite frequently found in the medfly (6 and 7 times, respectively), while only *β-tubulin* is found only once in the olive fly. Secondly, in quite a few occasions (indicated by the genes in bold in Table 2) the same HKGs are found in the top three genes in the same tissue of both insects. For example, in eggs *14-3-3zeta* and *RPL19* are ranked in the top three HKGs in both the medfly and the olive fly. All things being equal, the probability of finding one particular gene out of the nine tested HKGs among three selected genes is ⅓; the probability of finding two particular genes out of nine tested HKGs among three selected genes is ^1^∕_12_; while the probability of finding three particular genes out of nine tested HKGs among three selected genes is ^1^∕_84_ (calculation based on hypergeometric probabilities). Furthermore, the probability of finding the *same two* of the nine tested HKGs among three genes of both organisms (independent selections from a probability point of view) acquires the statistically significant value of 0.0069 (^1^∕_12_ × ^1^∕_12_), while finding the *same three* of the nine tested HKGs among three genes of both organisms acquires the statistically significant value of 0.00014 (^1^∕_84_ × ^1^∕_84_). This observation may suggest a biological explanation for the stability of *14-3-3zeta, RPL19* and *actin3* in heads or the stability of *14-3-3zeta* and *RPL19* in eggs of both species. A similar situation is detected in thoraces, MAGs and antennae, but not in FAGs or maxillary palps. Therefore, one can imagine that similar patterns of neuronal development in heads or embryonic development in eggs of both species would require similar expression of HKGs; on the contrary, differences in the female reproductive system (FAGs) or different diets (perceived by the maxillary palps) between the two insects would be reflected in the expression of different HKGs. More analyses are needed to substantiate such claims that are beyond the scope of this article.

Closing, we should iterate once again that the stability of common HKGs should not be taken for granted and that a lot of caution is needed in the choice of the appropriate HKGs. In fact, there is a need to validate the use of the proper HKG more often than practically encountered in recent literature. Even though we consider that our analysis offers a useful tool in the medfly and olive fly research community, we do encourage researchers to check these HKGs on their own subjects before use in a particular expression study.

## Methods

### Fly strains

The ‘Benakeion’ medfly and the ‘Demokritos’ olive fly strains were used in the experiments. The ‘Benakeion’ strain was originally established at the Benakeion Institute of Phytopathology, Athens, Greece, and has been kindly provided by Prof Nikos Papadopoulos at the Department of Agriculture Crop Production and Rural Environment, University of Thessaly, Greece. The ‘Demokritus’ strain originally comes from the Nuclear Research Centre in Athens, Greece, and has been reared in our laboratory for over 15 years. Both strains are maintained in wooden, nylon-screened, holding cages (30 × 30 × 30 cm) under an LD 14:10 h photocycle at 25 ± 1 °C and 60 ± 10% relative humidity. Olive fly rearing conditions are described in refs 63, 64, 65, while medfly conditions are described by Boller^66^.

### RNA isolation from specific tissues of *Ceratitis capitata* and *Bactrocera oleae*

Thirteen specific tissues at different developmental stages were used, as shown in Table 1. Eggs were collected from adult females 15 minutes after being laid. Larvae were 2^nd^ stage and pupae were harvested 12 hours after pupation. All dissected tissues (heads, thoraces and abdomens, as well as reproductive and olfactory) were from 5 day-old adult male and female insects.

Total RNA was isolated with the use of TRIsure™ (Bioline) following the instructions of the manufacturer with minor modifications. RNA extraction was followed by an additional DNA removal using the TURBO DNA-free Kit (Ambion-Invitrogen), according to manufacturer’s instructions. The integrity of RNA was assessed in a 1% agarose gel electrophoresis and quantified by Qubit^®^ 2.0 Fluorometer (Thermo Fisher Scientific).

The RNA extracted from: a single larva, a single pupa, a single head, a single thorax, a single abdomen and a set of ovaries from a single female, was quantified by Qubit and the amount of 2 μg was used for cDNA preparation. The entire RNA amount extracted from: a single egg, one pair of MAGs, one pair of testes, one pair of FAGs, and maxillary palps, antennae or ovipositors from a pool of 4 individual flies, was used for cDNA preparation, since the amount of RNA was undetectable.

DNA-free total RNA was converted into cDNA using 300 ng Random hexamer primers (equimolar mix of N_5_A, N_5_G, N_5_C and N_5_T), 200 units MMLV Reverse Transcriptase (Bioline), 10× reaction buffer, 40 mM dNTP mix and 40 units RNase Inhibitor (Bioline) according to the manufacturer’s instructions.

### Expression stability of candidate reference genes in *C. capitata* and *B. oleae*

82 to 150 bp amplicons from nine different housekeeping genes commonly used in other dipteran species were analyzed. The genes considered were: *RPL19 (ribosome protein L19*), *tbp (TATA-binding protein*), *ubx (ultrabithorax*), *GAPDH (glyceraldehyde 3-phosphate dehydrogenease*), *α-TUB (α-tubulin*), *β-TUB (β-tubulin*), *14-3-3zeta, RPE (RNA polymerase II*) and *actin3* (Supplementary Table S6). For the medfly, primers were based on sequences retrieved in the NCBI database. For the olive fly, primers were based on the sequences obtained during the transcriptome analysis of *B. oleae*^55^,^67^. Specific primers for the amplification of these HKGs were designed by Primer-BLAST^44^ (Supplementary Table S7). Each primer was also evaluated using OligoAnalyzer 3.1 tool^68^ in order to avoid hairpin formation and self-/hetero-dimerization of the oligonucleotides.

Relative quantitation was used to analyze changes in expression levels of the selected genes using a quantitative real-time PCR approach. The RT-qPCR conditions were: polymerase activation and DNA denaturation step at 95 °C for 4 min, followed by 40 cycles of denaturation at 95 °C for 30 s, annealing/extension and plate read at 56 °C (for all the tested housekeeping genes) and 60 °C (only for the reference genes *RPE* and *actin3*) for 30 s and finally, a step of melting curve analysis at a gradual increase of temperature over the range 55 °C → 95 °C. In this step, the detection of one gene specific peak and the absence of primer dimer peaks were assured. Each reaction was performed in a total volume of 15 μl, containing 5 μl from a 1:10 dilution of the cDNA template, 1 × iTaq Universal SYBR Green Supermix (Bio-Rad) and 400 nM of each primer. The reactions were carried out on Bio-Rad Real-Time thermal cycler CFX96 (Bio-Rad, Hercules, CA, USA) and data analyzed using the CFX Manager™ software. The expression of the reference genes was measured in 8 or 10 biological replicates, as indicated in Table 1. Three negative controls were also used. All reactions were done in triplicate (three technical replicates). The amplification efficiency of the reactions was calculated by the CFX Manager™ software (Bio-Rad). The PCR efficiency (E) and the correlation coefficient (R^2^) characterizing each standard curve are given in Supplementary Table S7. Efficiencies for all tested genes varied from 90.1% to 106.4%. The 2^−ΔΔCt^ method was used for the analysis of relative gene expression^69^.

#### *geNorm* analysis

The expression stability of the nine reference genes was assessed using the *geNorm* software. This algorithm is based on the principle that the logarithmically transformed expression ratio between two genes should be constant if both genes are stably expressed in a given sample set. The candidate reference genes were ranked by *geNorm* based on the expression stability value M, which is calculated for all genes under study. The lower the M value, the higher the gene’s expression stability. Furthermore, *geNorm* performs a stepwise calculation of the pairwise variation (V_n_/V_n+1_) between sequential normalization factors (NF_n_ and NF_n+1_) to determine the optimal number of reference genes required for accurate normalization^9^. Results are presented in Fig. 1 and Supplementary Table S1.

*Normfinder*^39^ is an algorithm for identifying the optimal normalization gene among a set of candidate genes. This software is based on a mathematical model of gene expression that enables estimation not only of the overall variation of the candidate normalization genes but also of the variation between samples subgroups of the sample set^39^. Results are presented in Supplementary Table S2A and S2B for *C. capitata* and *B. oleae*, respectively.

*BestKeeper* determines the most stably expressed genes based on the coefficient of correlation (r) to the *BestKeeper* Index (BI), which is the geometric mean of the candidate reference gene Cq values. Additionally, it calculates the standard deviation (SD) and the coefficient of variation (CV) based on the Cq values of all candidate reference genes^40^. Reference genes are identified as the most stable genes, i.e. those that exhibit the lowest coefficient of variance and standard deviation^70^. Results are presented in Supplementary Table S3A and S3B for *C. capitata* and *B. oleae*, respectively.

The *RefFinder* tool ranks all the potential reference genes according to the gene expression stability based on the rankings from *geNorm, Normfinder, BestKeeper* and the comparative ΔΔCt method programs. Also, this program assigns an appropriate weight to an individual gene and calculates the geometric mean of their weights for the overall final ranking^71^. Results are presented in Supplementary Table S4A and S4B for *C. capitata* and *B. oleae*, respectively.

### Statistical Analysis

Four different types of Microsoft Excel-based software, *geNorm*^9^, *NormFinder*^39^, *BestKeeper*^40^ and *RefFinder*^41^ were used to rank the expression stability of reference genes for all the experimental sets in the specific tissues of the medfly and the olive fruit fly. Relative quantities were used for *geNorm* and *NormFinder*, while *BestKeeper* analyses and the web-based program *refFinder* were based on untransformed Cq values. All four software packages were used according to the manufacturer’s instructions.

The consensus rank of the reference genes was estimated by the combination of the stability measurements obtained by *geNorm, Normfinder* and *BestKeeper*. More specifically, the raw scores calculated by these three software (M value by *geNorm*, stability value by *Normfinder* and SD by *BestKeeper*) were transformed into standard scores (z-score) for each housekeeping gene separately. The average of the three z-scores was subsequently calculated and the final rank was computed using the RANK function in Excel software. The above measurements were produced for every single reference gene in each one of the insect tissues under study. Thus, a consensus ranking of all nine genes was estimated for each one of the 13 tissues separately. Results are presented in Supplementary Table S5A and S5B for *C. capitata* and *B. oleae*, respectively.

### Ethics statement

The study was carried out on laboratory reared olive flies and medflies. No specific permissions are required for these experiments or collections, since these studies did not involve endangered or protected species.

## Additional Information

**How to cite this article**: Sagri, E. *et al*. Housekeeping in Tephritid insects: the best gene choice for expression analyses in the medfly and the olive fly. *Sci. Rep.* **7**, 45634; doi: 10.1038/srep45634 (2017).

**Publisher's note:** Springer Nature remains neutral with regard to jurisdictional claims in published maps and institutional affiliations.

## Supplementary Material

Supplementary Information

## Figures and Tables

**Table 1:** The thirteen tested tissues of *C. capitata* and *B. oleae*.

Tested tissues		Biological replicates
Developmental Stages	Egg	10 individuals
	Larva	10 individuals
	Pupa	10 individuals
Body parts	Head	10 individual parts (5 male and 5 female)
	Thorax	10 individual parts (5 male and 5 female)
	Abdomen	10 individual parts (5 male and 5 female)
Reproductive System	MAGs	10 pairs (1 pair of MAGs/fly)
	Testes	10 pairs (1 pair of testes/fly)
	FAGs	10 pairs (1 pair of FAGs/fly)
	Ovaries	10 sets (1 set of ovaries/fly)
	Ovipositors	8 pools (4 flies/pool)
Olfactory System	Maxillary palps	8 pools (4 flies/pool)
	Antennae	8 pools (4 flies/pool)

**Table 2:** Consensus ranking of tested *Ceratitis capitata* and *Bactrocera oleae* housekeeping genes according to the mean of the z-scores of their stability values obtained by *geNorm, NormFinder* and *BestKeeper*.

Tested tissues		The best ranking reference genes in *C. capitata*			The best ranking reference genes in *B. oleae*		
Developmental stages	Egg	***14-3-3 zeta***	***RPL19***	*β-TUB*	*RPE*	***14-3-3 zeta***	***RPL19***
	Larva	***RPE***	*actin3*	*RPL19*	*14-3-3zeta*	***RPE***	*GAPDH*
	Pupa	*tbp*	***RPL19***	*β-TUB*	***RPL19***	*14-3-3zeta*	*RPE*
Body tissues	Head	***14-3-3 zeta***	***RPL19***	***actin3***	***14-3-3zeta***	***RPL19***	***actin3***
	Thorax	*RPL19*	***GAPDH***	***14-3-3zeta***	***14-3-3zeta***	***GAPDH***	*β-TUB*
	Abdomen	*α-TUB*	***GAPDH***	*β-TUB*	*14-3-3zeta*	***GAPDH***	*ubx*
Reproductive system	Testes	***actin3***	*RPL19*	*α-TUB*	*14-3-3 zeta*	***actin3***	*RPE*
	MAGs	***RPL19***	*α-TUB*	***GAPDH***	***RPL19***	*actin3*	***GAPDH***
	Ovaries	***GAPDH***	*α-TUB*	*RPE*	*actin3*	***GAPDH***	*RPL19*
	FAGs	*β-TUB*	*tbp*	***RPE***	*GAPDH*	***RPE***	*actin3*
	Ovipositor	*β-TUB*	*α-TUB*	***14-3-3 zeta***	*tbp*	***14-3-3 zeta***	*RPL19*
Olfactory system	Antennae	***14-3-3 zeta***	*β-TUB*	***GAPDH***	***14-3-3 zeta***	*actin3*	***GAPDH***
	Maxillary palps	*α-TUB*	*RPL19*	*β-TUB*	*ubx*	*GAPDH*	*actin3*

Only the first three genes are indicated, listed from the most stable (left) to the least stable (right) gene order. Genes in bold contain highly ranked HKGs that are common in both *C. capitata* and *B. oleae*.
