# Supplementary material for: Housekeeping in Tephritid insects: the best gene choice for expression analyses in the medfly and the olive fly
Source: Sci Rep. 2017 Apr 3;7:45634. doi: 10.1038/srep45634 (PMC5377319; doi:10.1038/srep45634)
Supplement: Supplementary Information [file srep45634-s1.pdf]

Housekeeping in Tephritid insects: the best gene choice for expression analyses in the medfly and the olive fly

Efthimia Sagri[1], Panagiota Koskinioti[1], Maria-Eleni Gregoriou[1], Konstantina T Tsoumani[1], Yiannis C Bassiakos[2] and Kostas D Mathiopoulos[1§]

[1] Department of Biochemistry and Biotechnology, University of Thessaly, Larissa, Greece

[2]Department of Economic Sciences, National and Kapodistrian University of Athens, Athens, 10559, Greece

Supplementary Tables

Table S1. Suggested combination of *C. capitata* and *B. oleae* housekeeping genes that should be used for optimal normalization according to geNorm

Tissue	*Ceratitis capitata*		*Bactrocera oleae*	
	**Lowest Pairwise Variation value (V)**	**Suggested combination of HKG (from most stable to least stable)**	**Lowest Pairwise Variation value (V)**	**Suggested combination of HKG (from most stable to least stable)**
Egg	V3/4=0.155	14-3-3zeta, β-TUB, RPL19	V4/5=0.146	ubx, 14-3-3zeta, tbp, RPE
Larva	V2/3=0.073	α-TUB, 14-3-3zeta	V2/3=0.149	RPE, 14-3-3zeta
Pupa	V2/3=0.120	RPL19, tbp	V2/3=0.107	RPE, ubx
Head	V2/3=0.072	14-3-3zeta, actin3	V2/3=0.108	14-3-3zeta, GAPDH
Thorax	V2/3=0.085	tbp, α-TUB	V3/4=0.135	RPL19, 14-3-3zeta, ubx
Abdomen	V2/3=0.090	GAPDH, β-TUB	V3/4=0.154	α-TUB, 14-3-3zeta, GAPDH
MAGs	V3/4=0.116	14-3-3zeta, RPL19, α-TUB	V2/3=0.358	tbp, ubx
Testes	V2/3=0.078	RPL19, α-TUB	V3/4=0.157	14-3-3zeta, RPL19, α-TUB
Ovaries	V2/3=0.055	GAPDH, tbp	V2/3=0.122	RPE, GAPDH
FAGs	V2/3=0.123	GAPDH, β-TUB	V6/7=0.226	14-3-3zeta, RPL19, α-TUB, GAPDH, actin3, ubx*
Antennae	V3/4=0.477	GAPDH, β-TUB, RPL19	V2/3=0.309	actin3, RPL19
Maxillary palps	V3/4=0.150	α-TUB, β-TUB, actin3	V4/5=0.155	β-TUB, RPL19, ubx, GAPDH
Ovipositor	V4/5=0.198	α-TUB, actin3, β-TUB, 14-3-3zeta	V7/8=0.375	GAPDH, β-TUB, RPL19, 14-3-3zeta*, RPE*, α-TUB*, ubx*

*The M value of these genes is higher than the cutoff value of 1.5

Table S2A: Stability value of candidate reference genes in the various tissues of *Ceratitis capitata* evaluated by Normfinder

Rank	Egg		Larva		Pupa		Testes		MAGs		Ovaries		FAGs	
	Gene name	Stability value	Gene name	Stability value	Gene name	Stability value	Gene name	Stability value	Gene name	Stability value	Gene name	Stability value	Gene name	Stability value
1	*14-3-3zeta*	0.008	*RPE*	0.012	*tbp*	0.023	*α-TUB*	0.002	*GAPDH*	0.015	*GAPDH*	0.054	*β-TUB*	0.014
2	*β-TUB*	0.011	*actin 3*	0.025	*RPL19*	0.046	*RPL19*	0.003	*α-TUB*	0.097	*tbp*	0.065	*tbp*	0.032
3	*GAPDH*	0.015	*14-3-3 zeta*	0.029	*14-3-3 zeta*	0.117	*14-3-3 zeta*	0.008	*RPL19*	0.119	*RPL19*	0.115	*RPL19*	0.036
4	*RPL19*	0.015	*α-TUB*	0.040	*GAPDH*	0.141	*RPE*	0.008	*tbp*	0.123	*α-TUB*	0.115	*14-3-3 zeta*	0.041
5	*α-TUB*	0.031	*RPL19*	0.045	*β-TUB*	0.145	*β-TUB*	0.008	*14-3-3 zeta*	0.125	*RPE*	0.138	*RPE*	0.058
6	*tTbp*	0.047	*β-TUB*	0.053	*α-TUB*	0.198	*actin 3*	0.008	*RPE*	0.162	*14-3-3 zeta*	0.139	*ubx*	0.068
7	*ubx*	0.144	*ubx*	0.056	*RPE*	0.238	*GAPDH*	0.009	*β-TUB*	0.206	*actin 3*	0.221	*GAPDH*	0.097
8	*RPE*	0.156	*GAPDH*	0.073	*ubx*	0.248	*tbp*	0.020	*ubx*	0.235	*ubx*	0.287	*actin 3*	0.135
9	*actin3*	0.158	*tbp*	0.153	*actin 3*	0.396	*ubx*	0.032	*actin 3*	0.401	*β-TUB*	0.380	*α-TUB*	0.159

Table S2A (cont): Stability value of candidate reference genes in the various tissues of *Ceratitis capitata* evaluated by Normfinder

Rank	Head		Thorax		Abdomen		Antennae		Maxillary palps		Ovipositors	
	Gene name	Stability value	Gene name	Stability value	Gene name	Stability value	Gene name	Stability value	Gene name	Stability value	Gene name	Stability value
1	*14-3-3 zeta*	0.070	*GAPDH*	0.050	*GAPDH*	0.007	*14-3-3 zeta*	0.050	*RPL19*	0.065	*β-TUB*	0.024
2	*actin 3*	0.091	*RPL19*	0.052	*α-TUB*	0.012	*β-TUB*	0.054	*α-TUB*	0.066	*α-TUB*	0.026
3	*RPL19*	0.092	*14-3-3 zeta*	0.060	*β-TUB*	0.015	*RPL19*	0.076	*β-TUB*	0.075	*GAPDH*	0.045
4	*tbp*	0.104	*ubx*	0.062	*14-3-3 zeta*	0.019	*GAPDH*	0.122	*14-3-3 zeta*	0.087	*actin 3*	0.053
5	*α-TUB*	0.135	*actin 3*	0.069	*RPL19*	0.020	*α-TUB*	0.160	*actin 3*	0.090	*14-3-3 zeta*	0.066
6	*β-TUB*	0.172	*β-TUB*	0.072	*tbp*	0.037	*RPE*	0.188	*GAPDH*	0.096	*RPL19*	0.075
7	*RPE*	0.180	*α-TUB*	0.075	*RPE*	0.055	*actin 3*	-	*tbp*	0.135	*Tbp*	0.083
8	*GAPDH*	0.208	*tbp*	0.077	*actin 3*	0.082	*tbp*	-	*RPE*	0.208	*Ubx*	-
9	*ubx*	0.213	*RPE*	0.105	*ubx*	0.191	*ubx*	-	*ubx*	-	*RPE*	-

Table S2B: Stability value of candidate reference genes in the various tissues of *Bactrocera oleae* evaluated by Normfinder

	Egg		Larva		Pupa		Testes		MAGs		Ovaries		FAGs	
**Rank**	**Gene name**	**Stability value**	**Gene name**	**Stability value**	**Gene name**	**Stability value**	**Gene name**	**Stability Value**	**Gene name**	**Stability value**	**Gene name**	**Stability value**	**Gene name**	**Stability value**
1	*RPE*	0.022	*actin 3*	0.011	*RPE*	0.034	*14-3-3 zeta*	0.046	*RPL19*	0.017	*tbp*	0.022	*α-TUB*	0.040
2	*RPL19*	0.032	*RPE*	0.013	*α-TUB*	0.040	*RPL19*	0.052	*14-3-3 zeta*	0.029	*RPE*	0.032	*actin 3*	0.102
3	*α-TUB*	0.041	*tbp*	0.018	*RPL19*	0.047	*ubx*	0.055	*tbp*	0.040	*GAPDH*	0.053	*RPE*	0.102
4	*14-3-3 zeta*	0.042	*α-TUB*	0.019	*tbp*	0.054	*α-TUB*	0.059	*ubx*	0.051	*α-TUB*	0.053	*GAPDH*	0.113
5	*actin 3*	0.048	*ubx*	0.031	*actin 3*	0.078	*RPE*	0.097	*actin 3*	0.063	*RPL19*	0.060	*14-3-3 zeta*	0.121
6	*tbp*	0.073	*GAPDH*	0.035	*14-3-3 zeta*	0.082	*actin 3*	0.097	*RPE*	0.064	*ubx*	0.086	*RPL19*	0.122
7	*ubx*	0.079	*14-3-3 zeta*	0.075	*ubx*	0.086	*GAPDH*	0.100	*α-TUB*	0.081	*14-3-3 zeta*	0.106	*ubx*	0.127
8	*β-TUB*	0.140	*RPL19*	0.079	*GAPDH*	0.109	*tbp*	0.111	*β-TUB*	0.094	*actin 3*	0.119	*β-TUB*	0.141
9	*GAPDH*	0.177	*β-TUB*	0.089	*β-TUB*	0.170	*β-TUB*	0.147	*GAPDH*	0.095	*β-TUB*	0.178	*tbp*	0.184

Table S2B (cont): Stability value of candidate reference genes in in the various tissues of *Bactrocera oleae* evaluated by Normfinder

	Head		Thorax		Abdomen		Antennae		Maxillary palps		Ovipositors	
**Rank**	**Gene name**	**Stability value**	**Gene name**	**Stability value**	**Gene name**	**Stability value**	**Gene name**	**Stability value**	**Gene name**	**Stability value**	**Gene name**	**Stability value**
1	*14-3-3 zeta*	0.025	*RPL19*	0.030	*tbp*	0.052	*RPE*	0.001	*14-3-3 zeta*	0.017	*α-TUB*	0.058
2	*tbp*	0.042	*RPE*	0.032	*GAPDH*	0.098	*actin 3*	0.002	*RPE*	0.031	*ubx*	0.059
3	*α-TUB*	0.049	*β-TUB*	0.040	*α-TUB*	0.140	*14-3-3 zeta*	0.002	*ubx*	0.034	*GAPDH*	0.064
4	*ubx*	0.077	*α-TUB*	0.041	*14-3-3 zeta*	0.149	*α-TUB*	0.003	*α-TUB*	0.047	*RPL19*	0.066
5	*RPL19*	0.080	*ubx*	0.046	*RPL19*	0.157	*GAPDH*	0.005	*GAPDH*	0.053	*actin 3*	0.091
6	*β-TUB*	0.088	*tbp*	0.070	*ubx*	0.201	*RPL19*	0.015	*RPL19*	0.056	*RPE*	0.093
7	*GAPDH*	0.093	*14-3-3 zeta*	0.071	*actin 3*	0.257	*Ubx*	0.018	*actin 3*	0.103	*β-TUB*	0.128
8	*RPE*	0.095	*GAPDH*	0.092	*RPE*	0.266	*β-TUB*	0.090	*tbp*	0.138	*tbp*	0.199
9	*actin 3*	0.119	*actin 3*	0.095	*β-TUB*	0.412	*Tbp*	0.190	*β-TUB*	0.145	*14-3-3 zeta*	0.299

Table S3A: BestKeeper analysis results for the nine candidate reference gene in all the thirteen tested tissues of *Ceratitis capitata*

Gene name	Egg		Larva		Pupa		Head		Thorax		Abdomen		Testes		Ovaries		MAGs		FAGs		Antenna		Maxillary palps		Ovipositor	
	SD	CV	SD	CV	SD	CV	SD	CV	SD	CV	SD	CV	SD	CV	SD	CV	SD	CV	SD	CV	SD	CV	SD	CV	SD	CV
14-3-3 zeta	**1.93**	**5.89**	0.54	2.86	1.05	5.48	0.33	1.56	0.43	2.05	0.58	3.03	2.15	7.37	0.26	1.29	2.69	10.61	1.21	4.80	0.70	2.02	1.24	3.64	1.09	3.20
ubx	-	-	0.53	1.99	1.19	4.32	1.30	3.89	0.44	1.55	0.42	1.62	-	-	0.69	2.07	1.63	5.22	1.64	4.61	-	-	-	-	-	-
tbp	1.99	5.78	0.88	3.46	0.82	3.26	0.34	1.27	0.46	1.70	0.72	2.90	2.24	6.57	0.25	1.10	2.09	6.71	1.12	3.61	-	-	-	-	1.98	5.21
β-TUB	2.09	6.91	0.66	3.87	0.58	3.31	0.29	1.43	0.50	2.65	0.47	2.64	2.28	8.17	0.16	0.95	2.57	10.31	1.32	5.84	0.71	2.05	1.57	4.64	**0.99**	**3.04**
GAPDH	2.41	7.91	0.73	4.56	0.90	5.11	**0.22**	**1.13**	**0.25**	**1.52**	0.46	2.55	2.14	7.28	0.16	0.90	2.52	10.85	1.21	5.34	**0.58**	**1.74**	1.89	5.81	1.60	4.93
actin 3	-	-	0.39	2.31	1.42	7.07	0.41	1.79	0.47	2.48	1.26	6.01	**1.98**	**5.60**	0.34	1.33	2.75	8.78	1.49	6.26	-	-	1.40	3.93	1.51	4.26
RPE	-	-	0.43	1.76	0.77	3.23	0.42	1.54	0.48	1.81	1.01	4.17	2.32	6.73	**0.12**	**0.50**	1.14	3.86	1.70	5.31	-	-	-	-	-	-
RPL19	1.93	6.13	**0.31**	**1.81**	0.79	4.49	0.28	1.29	0.38	1.83	0.43	2.35	2.16	7.25	0.23	1.22	**0.85**	**3.46**	1.40	5.71	0.81	2.36	**1.17**	**3.56**	1.46	4.15
α-TUB	2.09	6.33	0.52	2.91	**0.53**	**2.97**	0.39	1.80	0.51	2.53	**0.35**	**1.82**	2.21	8.27	0.12	0.60	0.88	3.46	**1.02**	**3.97**	1.40	3.91	1.33	3.90	1.26	3.72

Notes: 1. The lowest SD values are indicated in bold, 2. Cells with no value (-) indicate HKGs that had no detectable expression in those tissues.

Table S3B: BestKeeper analysis results for the nine candidate reference gene in all the thirteen tested tissues of *Bactrocera oleae*

Gene name	Egg		Larva		Pupa		Head		Thorax		Abdomen		Testes		Ovaries		MAGs		FAGs		Antenna		Maxillary palps		Ovipositor	
	SD	CV	SD	CV	SD	CV	SD	CV	SD	CV	SD	CV	SD	CV	SD	CV	SD	CV	SD	CV	SD	CV	SD	CV	SD	CV
14-3-3 zeta	0.8	3.04	2.15	9.47	0.87	4.07	0.43	2.13	1.94	6.97	1.21	5.96	**0.34**	**1.61**	1.75	8.33	2.50	8.19	1.88	6.44	1.80	6.06	**1.94**	**6.23**	2.01	5.93
ubx	0.63	2.15	1.39	5.23	0.96	3.73	2.74	12.78	1.52	4.89	0.71	2.90	1.52	8.59	0.91	3.98	0.93	2.83	1.07	3.32	2.38	7.02	3.00	9.18	1.38	3.98
tbp	0.65	2.34	1.04	4.09	0.94	3.55	**0.28**	**1.17**	1.37	4.25	0.78	3.81	1.58	4.89	**0.67**	**3.17**	2.01	6.79	1.17	5.05	3.58	15.09	2.68	8.59	2.45	10.39
β-TUB	1.24	3.62	2.83	9.01	1.19	4.13	0.48	1.64	2.30	8.90	2.84	12.02	2.3	8.9	1.95	7.61	1.18	3.35	1.90	6.70	1.96	5.38	2.89	10.18	2.35	7.72
GAPDH	3.04	10.88	1.94	9.35	1.93	3.8	0.44	2.39	2.13	7.34	0.79	4.23	2.13	7.34	0.70	3.99	2.62	8.69	1.37	4.73	2.11	7.01	2.91	10.79	2.35	7.33
actin 3	1.13	3.19	1.24	6.09	1.17	4.94	1.49	7.42	2.52	7.87	1.06	5.90	2.52	7.87	1.95	8.53	2.14	7.57	1.52	5.75	1.63	5.44	2.65	8.96	2.66	7.75
RPE	**0.56**	**2.4**	**1.04**	**4**	0.55	2.6	0.37	1.51	**0.34**	**1.61**	1.60	6.73	1.37	4.25	1.48	6.61	0.80	2.44	1.54	4.67	**1.50**	**4.39**	2.35	6.98	1.51	4.27
RPL19	0.85	3.86	1.84	8.98	0.6	2.96	0.45	2.27	1.84	6.74	1.00	5.50	1.46	5.24	0.68	4.24	**0.28**	**1.35**	1.62	5.83	1.88	6.33	2.88	10.99	2.73	8.42
α-TUB	0.81	3.02	1.79	8.19	**0.12**	**0.55**	0.51	2.47	1.63	6.70	**0.51**	**2.54**	1.63	6.7	0.83	4.29	1.87	6.46	**0.96**	**2.66**	1.88	5.78	3.02	10.70	**1.09**	**3.02**

Notes: 1. The lowest SD values are indicated in bold.

Table S4A: Geomean of ranking values of candidate reference genes in the thirteen tested tissues of *Ceratitis capitata* evaluated by refFinder

Rank	Egg		Larvae		Pupae		Testes		MAGS		Ovaries		FAGS	
	Gene name	Geomean of ranking values	Gene name	Geomean of ranking values	Gene name	Geomean of ranking values	Gene name	Geomean of ranking values	Gene name	Geomean of ranking values	Gene name	Geomean of ranking values	Gene name	Geomean of ranking values
1	*tbp*	1.57	*α-TUB*	1.78	*tbp*	1.5	*14-3-3zeta*	1.57	*GAPDH*	1.57	*tbp*	1.68	*α-TUB*	1.57
2	*α-TUB*	2.11	*14-3-3zeta*	2.21	*14-3-3zeta*	2.55	*GAPDH*	1.86	*14-3-3zeta*	2	*RPL19*	2.34	*GAPDH*	1.68
3	*RPL19*	2.74	*RPL19*	2.99	*RPL19*	2.91	*tbp*	2.59	*α-TUB*	2.45	*14-3-3zeta*	2.91	*RPL19*	2.94
4	*GAPDH*	2.91	*RPE*	3.03	*β-tub*	3.36	*RPL19*	2.83	*RPL19*	2.78	*RPE*	3.46	*14-3-3zeta*	3.87
5	*β-tub*	4.53	*actin3*	4.16	*α-TUB*	3.98	*α-TUB*	4.73	*tbp*	5.14	*α-TUB*	3.83	*β-tub*	4.74
6	*14-3-3zeta*	5.48	*β-tub*	5.05	*RPE*	5.01	*actin3*	6.24	*β-tub*	5.96	*GAPDH*	4.33	*actin3*	5.09
7	*RPE*	6.48	*ubx*	5.66	*GAPDH*	5.69	*RPE*	6.74	*RPE*	6.48	*actin3*	6.44	*tbp*	5.86
8	*actin3*	8	*GAPDH*	8.24	*ubx*	8	*β-tub*	8	*ubx*	8	*β-tub*	8	*RPE*	8
9	*ubx*	-	*tbp*	8.74	*actin3*	9	*ubx*	9	*actin3*	9	*ubx*	9	*ubx*	9

Rank	Head		Thorax		Abdomen		Antennae		Maxillary palps		Ovipositors	
	Gene name	Geomean of ranking values	Gene name	Geomean of ranking values	Gene name	Geomean of ranking values	Gene name	Geomean of ranking values	Gene name	Geomean of ranking values	Gene name	Geomean of ranking values
1	*RPL19*	1.32	*RPL19*	1	*GAPDH*	1.32	*14-3-3zeta*	1.86	*β-tub*	1.41	*α-TUB*	1.41
2	*α-TUB*	2	*ubx*	1.86	*α-TUB*	2.21	*α-TUB*	2	*RPL19*	1.86	*GAPDH*	2.11
3	*tbp*	3.41	*actin3*	3.71	*RPL19*	2.78	*GAPDH*	2.28	*α-TUB*	2.99	*RPL19*	3.13
4	*RPE*	3.83	*GAPDH*	4.56	*14-3-3zeta*	3.5	*RPL19*	2.38	*GAPDH*	3.22	*14-3-3zeta*	3.22
5	*actin3*	3.98	*β-tub*	4.68	*β-tub*	3.94	*β-tub*	5	*tbp*	3.98	*tbp*	3.34
6	*GAPDH*	4.43	*14-3-3zeta*	4.73	*tbp*	6.24	*RPE*	6	*14-3-3zeta*	6	*actin3*	6
7	*14-3-3zeta*	7	*α-TUB*	7.24	*RPE*	7.24	*ubx*	-	*RPE*	7	*β-tub*	7
8	*β-tub*	8	*RPE*	7.35	*ubx*	7.35	*tbp*	-	*actin3*	8	*RPE*	-
9	*ubx*	9	*tbp*	8.24	*actin3*	8.24	*actin 3*	-	*ubx*	-	*ubx*	-

Table S4B: Geomean of ranking values of candidate reference genes in the thirteen tested tissues of *Bactrocera oleae* evaluated by refFinder

Rank	Egg		Larvae		Pupae		Testes		MAGS		Ovaries		FAGS	
	Gene name	Geomean of ranking values	Gene name	Geomean of ranking values	Gene name	Geomean of ranking values	Gene name	Geomean of ranking values	Gene name	Geomean of ranking values	Gene name	Geomean of ranking values	Gene name	Geomean of ranking values
1	*tbp*	1.732	*RPE*	2.59	*β-tub*	1.32	*tbp*	1.32	*Ubx*	1.5	*GAPDH*	2	*tbp*	1.86
2	*RPL19*	2.213	*β-tub*	2.63	*GAPDH*	2.21	*GAPDH*	2.34	*β-tub*	2.45	*actin3*	2.21	*GAPDH*	2
3	*actin3*	3.253	*actin3*	2.63	*tbp*	2.45	*β-tub*	3.13	*GAPDH*	3.13	*14-3-3zeta*	3.2	*RPL19*	2.78
4	*RPE*	3.5	*GAPDH*	2.78	*RPL19*	2.83	*RPE*	4.12	*14-3-3zeta*	3.66	*RPE*	3.83	*14-3-3zeta*	3.16
5	*α-TUB*	3.834	*tbp*	3.08	*actin3*	5.48	*RPL19*	4.3	*α-TUB*	3.94	*β-tub*	4.16	*β-tub*	4.21
6	*GAPDH*	4.949	*RPL19*	3.94	*RPE*	6.19	*14-3-3zeta*	5.44	*RPL19*	3.98	*RPL19*	4.3	*ubx*	4.74
7	*β-tub*	5.118	*14-3-3zeta*	7.45	*ubx*	6.19	*actin3*	5.66	*Tbp*	6.44	*tbp*	4.36	*actin3*	6.96
8	*ubx*	8	*ubx*	8	*14-3-3zeta*	8	*ubx*	6.45	*RPE*	8	*ubx*	8.24	*RPE*	7.44
9	*14-3-3zeta*	9	*α-TUB*	8.45	*α-TUB*	9	*α-TUB*	9	*actin3*	9	*α-TUB*	8.74	*α-TUB*	9

Rank	Head		Thorax		Abdomen		Antennae		Maxillary palps		Ovipositors	
	Gene name	Geomean of ranking values	Gene name	Geomean of ranking values	Gene name	Geomean of ranking values	Gene name	Geomean of ranking values	Gene name	Geomean of ranking values	Gene name	Geomean of ranking values
1	*14-3-3zeta*	1.73	*tbp*	2.38	*RPE*	1.41	*ubx*	1.32	*GAPDH*	1.5	*GAPDH*	1.57
2	*actin3*	2.21	*RPL19*	2.45	*β-tub*	1.57	*β-tub*	1.57	*14-3-3zeta*	2.21	*β-tub*	2
3	*RPE*	3.08	*ubx*	2.66	*tbp*	2.28	*tbp*	2.91	*RPL19*	3.98	*14-3-3zeta*	2.21
4	*tbp*	3.46	*GAPDH*	2.71	*RPL19*	4	*α-TUB*	4.28	*tbp*	4.16	*α-TUB*	2.91
5	*ubx*	3.76	*β-tub*	2.78	*14-3-3zeta*	5	*GAPDH*	4.47	*β-tub*	4.3	*tbp*	5.23
6	*GAPDH*	4.92	*14-3-3zeta*	5.38	*GAPDH*	6	*RPL19*	5.48	*ubx*	4.56	*ubx*	5.73
7	*β-tub*	5.6	*α-TUB*	6.96	*ubx*	7.48	*14-3-3zeta*	6.74	*RPE*	5.03	*RPL19*	7
8	*RPL19*	8	*RPE*	7.74	*α-TUB*	7.48	*RPE*	8	*α-TUB*	6.45	*RPE*	8
9	*α-TUB*	9	*actin3*	9	*actin3*	9	*actin3*	9	*actin3*	8.74	*actin3*	9

Table S5A: Consensus ranking of tested *Ceratitis capitata* housekeeping genes according to the mean of the z-scores of their stability values obtained by geNorm, NormFinder and BestKeeper. Candidate genes are listed from the most stable to the least stable gene order.

**Developmental stages**			**Body parts**			**Reproductive system**					**Olfactory system**	
Egg	Larva	Pupa	Head	Thorax	Abdomen	Testes	MAGs	Ovaries	FAGs	Ovipositors	Antennae	Maxillary Palps
14-3-3zeta	*RPE*	*tbp*	*14-3-3zeta*	*RPL19*	*α-TUB*	*actin3*	*RPL19*	*GAPDH*	*β-TUB*	*β-TUB*	*14-3-3zeta*	*α-TUB*
RPL19	*actin3*	*RPL19*	*RPL19*	*GAPDH*	*GAPDH*	*RPL19*	*α-TUB*	*α-TUB*	*tbp*	*α-TUB*	*β-TUB*	*RPL19*
β-TUB	*RPL19*	*β-TUB*	*actin3*	*14-3-3zeta*	*β-TUB*	*α-TUB*	*GAPDH*	*RPE*	*RPE*	*14-3-3zeta*	*GAPDH*	*β-TUB*
GAPDH	*14-3-3zeta*	*α-TUB*	*tbp*	*tbp*	*RPL19*	*14-3-3zeta*	*RPE*	*tbp*	*GAPDH*	*actin3*	*RPL19*	*actin3*
tbp	*α-TUB*	*14-3-3zeta*	*α-TUB*	*ubx*	*14-3-3zeta*	*GAPDH*	*Tbp*	*RPL19*	*14-3-3zeta*	*GAPDH*	*α-TUB*	*14-3-3zeta*
α-TUB	*β-TUB*	*GAPDH*	*GAPDH*	*β-TUB*	*tbp*	*β-TUB*	*14-3-3zeta*	*β-TUB*	*RPL19*	*RPL19*	-	*GAPDH*
RPE	*ubx*	*RPE*	*β-TUB*	*α-TUB*	*RPE*	*RPE*	*Ubx*	*14-3-3zeta*	*α-TUB*	-	-	-
actin3	*GAPDH*	*ubx*	*RPE*	*actin3*	*ubx*	*tbp*	*β-TUB*	*actin3*	*actin3*	-	-	-
ubx	*tbp*	*actin3*	*ubx*	*RPE*	*actin3*	*ubx*	*actin3*	*ubx*	*ubx*	-	-	-

Table S5B: Consensus ranking of tested *Bactrocera oleae* housekeeping genes according to the mean of the z-scores of their stability values obtained by geNorm, NormFinder and BestKeeper. Candidate genes are listed from the most stable to the least stable gene order.

**Developmental stages**			**Body parts**			**Reproductive system**					**Olfactory system**	
Egg	Larva	Pupa	Head	Thorax	Abdomen	Testes	MAGs	Ovaries	FAGs	Ovipositors	Antennae	Maxillary palps
RPE	*14-3-3zeta*	*RPL19*	*14-3-3zeta*	*14-3-3zeta*	*14-3-3zeta*	*14-3-3zeta*	*RPL19*	*actin3*	*GAPDH*	*tbp*	*14-3-3zeta*	*Ubx*
14-3-3zeta	*RPE*	*14-3-3zeta*	*RPL19*	*GAPDH*	*GAPDH*	*actin3*	*actin3*	*GAPDH*	*RPE*	*14-3-3zeta*	*actin3*	*GAPDH*
RPL19	*GAPDH*	*RPE*	*actin3*	*β-TUB*	*ubx*	*RPE*	*GAPDH*	*RPL19*	*actin3*	*RPL19*	*GAPDH*	*actin3*
Ubx	*tbp*	*GAPDH*	*RPE*	*RPE*	*RPE*	*GAPDH*	*14-3-3zeta*	*14-3-3zeta*	*tbp*	*ubx*	*RPL19*	*RPE*
Tbp	*α-TUB*	*actin3*	*ubx*	*actin3*	*actin3*	*tbp*	*tbp*	*RPE*	*RPL19*	*actin3*	*tbp*	*RPL19*
α-TUB	*ubx*	*tbp*	*tbp*	*RPL19*	*RPL19*	*α-TUB*	*RPE*	*α-TUB*	*14-3-3zeta*	*GAPDH*	*ubx*	*β-TUB*
actin3	*actin3*	*ubx*	*β-TUB*	*α-TUB*	*tbp*	*ubx*	*ubx*	*tbp*	*ubx*	*RPE*	*RPE*	*14-3-3zeta*
β-TUB	*RPL19*	*α-TUB*	*GAPDH*	*ubx*	*α-TUB*	*RPL19*	*α-TUB*	*ubx*	*α-TUB*	*β-TUB*	*α-TUB*	*tbp*
GAPDH	*β-TUB*	*β-TUB*	*α-TUB*	*tbp*	*β-TUB*	*β-TUB*	*β-TUB*	*β-TUB*	*β-TUB*	*α-TUB*	*β-TUB*	*α-TUB*

Table S6. Names and gene IDs of *C. capitata* and *B. oleae* genes. The names used in this study were slightly modified in order to maintain consistency with *Drosophila melanogaster* nomenclature.

Gene name used in the study	*Ceratitis capitata*		*Bactrocera oleae*	
	C. capitata gene name	Gene ID	*B. oleae* gene name	Gene ID
14-3-3zeta	14-3-3 protein zeta	101462599	14-3-3 protein zeta	106626490
α-TUB	tubulin alpha-1 chain (TBA1)	101459628	tubulin alpha-1 chain (TBA1)	106627729
β-TUB	tubulin beta-1 chain (TBB1)	101456544	tubulin beta-2 chain	106616149
tbp	TATA-box-binding protein (Tbp)	101456465	transcription initiation factor TFIID subunit 13	106624711
ubx	homeotic protein ultrabithorax (ubx)	101462158	homeotic protein ultrabithorax (ubx)	106614415
GAPDH	glyceraldehyde-3-phosphate dehydrogenase 2	105664420	glyceraldehyde-3-phosphate dehydrogenase 2	106614277
RPE	DNA-directed RNA polymerase II subunit RPB1	101449324	DNA-directed RNA polymerase II subunit RPB1	106626103
actin3	actin-3, muscle-specific	101462235	actin-3, muscle-specific	105229889
Rpl19	60S ribosomal protein L19 (RL19)	101460909	60S ribosomal protein L19 (RL19)	106622801

Table S7. Primer sequences used for the 9 housekeeping genes tested in *Ceratitis capitata* and *Bactrocera oleae*

Gene name	*Ceratitis capitata*				*Bactrocera oleae*			
	Primer pair	Amplicon size (bp)	Efficiency (E)	R^2	Primer pair	Amplicon size (bp)	Efficiency (E)	R^2
14-3-3zeta	F=GGTCTAGCACTAAACTTTTC<br>R=TGAGTCTTTGTATGAGTCC	138	93.4%	1.0	F=GTCTTCTCGACAAACACC<br>R=CCACCTCAGCTAAATACC	102	99.8%	1.0
α-TUB	F=GGTGCCCTACCCACGTATTC<br>R=ACCATCTGGTTGGCTGGTTC	132	91.6%	0.999	F=TACATGGCCTGTTGTATG<br>R=GCTTGGTCTTGATGGTAG	82	104.6%	0.995
β-TUB	F=TCTCTACCAGTTGATGCAC<br>R=CCGACAGAATAATGAACAC	105	96.3%	0.993	F=GCTCTTTACGACATCTGC<br>R=CACAGCCAACTTTCGTAG	150	99.8%	0.948
tbp	F=AATGGATCAGATGTTGAGTC<br>R=GTTTAGATCGTGTGTGTCTG	133	102.5%	0.951	F=GCTTCGATGTATGATGTATG<br>R=GATCTCCATTGCTTTGTG	121	90.1%	0.998
ubx	F=ACTAATGGACAGAACAATCC<br>R=ACTACCACCAGAAGTGTCC	102	99.9%	0.999	F=AGCAGCTATCTTAGAATGACC<br>R=TATAACCAACGCTTCAGG	91	99.9%	0.999
GAPDH	F=ATGAAGGTCGTATCTAATGC<br>R=TAGTTGCGTGAACAGTAGTC	115	94.1%	0.999	F=GGTGTCTTTACAACAATCG<br>R=TAGATACGACCTTCATGTCAG	148	98.8%	0.996
RPE	F=GATGAAAGTACTCTCCGAAC<br>R=TGTAACACAGCATTCATCTC	132	98.4%	0.996	F=TGTGGCAATGTGCGCTTACGG<br>R=AGGCTATGGTGGTGTCGGGTTC	127	98.7%	0.994
*actin3 **	F=CCACCAGAACGTAAATACTC<br>R=TCTCATTGAGCGTTTAGAAG	148	101.1%	0.998	F=GGTCGGTATGGGACAGAAGG<br>R=CTCACGATTGGCTTTTGGAT	220	106.4%	0.996
Rpl19	F=AACAAACGTGTACTGATGG<br>R=CACGTACTTTATGTCGTCTG	103	90.6%	0.999	F=CTTCACGTACTTTATGCCTTC<br>R=GCAAGGGTAATGTGTTCAA	126	97.3%	0.999

* *actin3:* for *Bactrocera oleae*, the primers were designed according to Shen et al., 2010
